# Gender balance in infectious diseases and hospital epidemiology journals

**DOI:** 10.1017/ash.2023.481

**Published:** 2023-10-27

**Authors:** Aldo Barajas-Ochoa, Manuel Ramirez-Trejo, Paloma Gradilla-Magaña, Aditee Dash, Jillian Raybould, Gonzalo Bearman

**Affiliations:** 1 Division of Infectious Diseases, Department of Medicine, Virginia Commonwealth University, Richmond, VA, 23298, USA; 2 Centro Universitario de Ciencias de la Salud, Universidad de Guadalajara, Guadalajara, México

## Abstract

**Objective:**

Diversity is recognized as a driver of excellence and innovation. Women represent a significant part of the infectious diseases (ID) and hospital epidemiology (HE) workforce. We aimed to assess gender representation among editors of top ID and HE journals and explore potential correlations with the gender of first and last authors in published articles.

**Methods:**

Using Scimago Journal & Country Rank, we identified 40 ID and 4 HE high-ranking journals. Editorial members were categorized by decision-making influence (levels I-III). We retrieved names of first and corresponding authors from 12 ID-focused journals’ 2019 research articles. Gender assignment for editors, first authors, and last authors utilized digital galleries and manual searches.

**Results:**

Among 2,797 editors from 44 journals, 33% were women. Female representation varied across editorial levels: 26% at level I, 36% at level II, and 31% at level III. Gender balance disparities existed among journals. Female first authors accounted for 50%, and female last authors accounted for 36% of the 2,725 published articles. We found weak but significant correlations between the editors’ gender and the gender of the first and last authors.

**Conclusion:**

Gender representation among ID and HE journal editors displayed unevenness, but no overt vertical segregation was observed. A generational transition among authors may be underway. Our findings suggest that a generational transition may be occurring among authors.

## Introduction

Over the past two decades, the representation of women in the field of medicine has undergone dynamic changes in various countries.^1,2^ For instance, in the United States of America (USA), there has been a notable increase in the proportion of female physicians. In 2007, women accounted for 28% of active physicians and 45% of residents and fellows. Fast forward to 2021, these numbers increased to 37.1% and 47.3% for active physicians and residents/fellows, respectively.^2^ Similarly, the landscape in infectious diseases (ID) has also transformed.^2,3^ In 2007, the USA had one ID physician for every 46,952 individuals, with 33% of the 6,424 active ID physicians being women and a majority falling below the age of 55. Fourteen years later, the number of ID physicians increased to 9,913, with 1 physician for every 32,944 people, and the proportion of women rose to 43%.^2^ However, the representation of younger individuals (under 55) in this field declined.

Given the growth of ID specialists, one might expect an increase in female leadership positions. However, studies conducted during different periods do not support this assumption. For instance, from 2008 to 2017, only 20% of the presidents of the Infectious Diseases Society of America (IDSA) were women.^4^ Gender disparities were also observed in achieving the rank of full professor in academic ID in 2016,^5^ as well as in compensation in 2015,^6^ and an underrepresentation of female authors on IDSA guidelines’ editorial boards for prominent ID publications from 2004 to 2018.^7^

The influence of editorial bodies in scientific publishing is pivotal, as journal editors act as gatekeepers to science, influencing research trends and significantly impacting the professional advancement of individuals, thus shaping academic and research programs.^8^ Yet, these editorial bodies have also been found to exhibit gender imbalances. A recent study across 41 international medical journal categories found that only 21% of editors-in-chief were women, with significant variability among medical specialties. In ID, this percentage was only 10% in 2019.^9^ Another recent study revealed that only 22% of editors-in-chief of ID and microbiology journals were women.^10^

With women comprising an increasing proportion of the ID and Hospital Epidemiology (HE) workforce, it becomes pertinent to assess the gender balance of editors and authors in leading ID and HE journals. This study’s primary objective is to evaluate the gender representation of editors and editorial boards in leading ID- and HE-focused journals in 2023. The study also assesses the gender distribution of the first and last authors of a convenience sample of original articles published in 2019 and explores if there is a correlation between the gender of editors and authors.

## Methods

This study uses a cross-sectional audit-type design and it was conducted between November 2022 and March 2023.

### Identification, selection, and categorization of editorial members

We evaluated top ID-focused journals listed in the first quartile of Scimago Journal & Country Rank^11^ and added a convenience sample of HE journals we had access to. The full names and positions of editorial members were extracted from the websites of each selected journal. If the given name was not noted, it was searched in Google Scholar.

We categorized the positions of editorial members based on their presumed influence on a manuscript’s fate to enrich the assessment of gender representation. However, due to the lack of uniformity among journals regarding editorial position labels and the function performed by members in these positions, we developed a spreadsheet containing the specific labels of the entire editorial group. Subsequently, we categorized these positions based on their presumed influence on manuscript outcomes. Positions labeled as level I, including editor-in-chief and deputy editor, were considered to have the highest decision-making power. Level II positions, such as associate editor, feature editor, and section editor, had relatively less influence as they were responsible for fewer manuscripts. Level III positions, such as advisory, board members, and editorial board members, reviewed only a few manuscripts per year and thus had less influence. Administrative or honorary positions, such as editor emeritus, managing editor, assistant editor, and consulting editor, were classified as level IV and were excluded from our analysis.

### Exploratory variables

The first exploratory variable aimed to assess whether the country of affiliation of the editors was associated with gender distribution. Countries with at least 34 editors were individually categorized, while the remaining countries were grouped under the “rest of the world.”

The second exploratory variable assessed if there was an association between the gender of editorial members, stratified by their level of influence, and the acceptance of articles based on the gender of the first or last author. Based on preliminary results, we selected a convenience sample of 12 journals demonstrating distinct distributions of male/female editorial members, with imbalances skewed toward men, roughly balanced, or skewed toward women. We accessed the original articles published in 2019 from Web of Science for these selected journals, utilizing an institutional account. The year 2019 was chosen as a representative year to minimize potential confounding factors associated with the SARS-CoV-2 pandemic. We registered the names and affiliations of all first and last authors, with particular attention to the given names of the authors. In cases where citation information only provided last names and initials, a manual search was conducted using available names and affiliations on platforms such as Google Scholar and LinkedIn.

The third exploratory variable aimed to evaluate whether the country of affiliation of the first and last authors was associated with the authors’ gender and their preferred journals for publication. Only the countries with the most published articles among the previously mentioned 12 selected journals were considered, while the remaining countries were grouped as “rest of the world.”

### Gender guessing and assignment

To infer gender and assign it to individuals, we followed established methodologies outlined in previous research.^12–15^ Our approach prioritized accuracy above other considerations.^16^ Gender was classified as female or male (binary) based on the cultural association of a given name with a woman or a man, respectively. To accomplish this, we utilized a digital gallery available at https://gender-api.com/ and assigned gender based on the returned result. We set a threshold of at least 100 samples with an accuracy rate of at least 80% to ensure robustness.^17^ When the estimated accuracy for a specific gender denomination did not meet these parameters, individuals with such names were manually searched using their full names and affiliations on academic platforms like Google Scholar and LinkedIn. We maintained the misclassification rate of names through these confidence measures at or below 5%.^17^

### Statistical analysis

Our analysis is grounded on 2 assumptions. First, we consider all editorial members as one system and examine the numbers and proportions of males and females within this system, categorized by decision levels, to evaluate gender balance. Second, we assume that the editorial members of each journal should be treated as independent, heterogeneous, and non-comparable populations. This assumption is supported by evident disparities such as variations in editorial membership selection committees, the timing of membership, job descriptions, labels, the specific scope of each journal, and even the publisher. Hence, with this assumption, we recognize the independent distribution of gender within each individual journal, thereby precluding statistical comparisons between them.

All variables examined in this study were of nominal nature, including editor and author gender, journal names, editor’s decision level, and country of affiliation. To assess independence, we utilized the Pearson chi-square test (χ2), and for determining the strength of associations in contingencies greater than 2 × 2, we used phi or Cramér’s V.^18^ We considered the strength of association to be weak when it fell between 0.1 and 0.3, medium when between 0.4 and 0.5, and strong when exceeding 0.5. The 2-tailed significance level was set at *P* ≤ 0.05.

## Results

Forty ID-focused and 4 HE-focused journals were included. Overall, there were 2,797 editor names, but 11 were further excluded due to missing data. In total, 2,786 editor names from 44 journals were analyzed. Table 1 shows the frequency distribution of editorial members by journal, gender, and level of decision as of March 21, 2023.

**Table 1.** Distribution of editorial members by journal, decision level, and gender

Journal ranking*	Journal name	Editorial members by decision level		
		I	II	III
1	Immunity	5	0	0
	Female, *n* (%)	3 (60)	–	–
2	The Lancet Microbe	1	0	9
	Female, *n* (%)	0	–	7 (78)
3	The Lancet Infectious Diseases	1	1	25
	Female, *n* (%)	1 (100)	0	14 (56)
4	Nature Reviews Microbiology	1	0	1
	Female, *n* (%)	0	–	1 (100)
5	Clinical Microbiology Reviews	1	7	34
	Female, *n* (%)	0	4 (57)	14 (41)
6	The Lancet HIV	1	1	1
	Female, *n* (%)	0	1 (100)	0
7	Journal of Infection	2	9	4
	Female, *n* (%)	0	1 (11)	0
8	Clinical Infectious Diseases	1	23	209
	Female, *n* (%)	0	10 (43)	52 (25)
9	Microbiology and Molecular Biology Reviews	1	12	1
	Female, *n* (%)	1 (100)	6 (50)	1 (100)
10	FEMS Microbiology Reviews	3	34	0
	Female, *n* (%)	1 (33)	10 (29)	–
11	Drug Resistance Updates	1	4	61
	Female, *n* (%)	0	1(25)	10 (16)
12	Emerging Infectious Diseases	2	25	39
	Female, *n* (%)	0	3 (12)	10 (26)
13	Journal of Travel Medicine	1	24	42
	Female, *n* (%)	1 (100)	7 (29)	8 (19)
14	Trends in Microbiology	1	0	24
	Female, *n* (%)	0	–	12 (50)
15	Journal of Clinical Virology	2	16	34
	Female, *n* (%)	0	5 (31)	15 (44)
16	Emerging Microbes and Infections	2	6	51
	Female, *n* (%)	0	0	10 (20)
17	Clinical Microbiology and Infection	1	20	0
	Female, *n* (%)	0	8 (40)	–
18	Cellular and Molecular Immunology	3	7	95
	Female, *n* (%)	0	0	16 (16)
19	npj Vaccines	1	10	82
	Female, *n* (%)	0	2 (20)	21 (26)
20	Journal of Medical Virology	1	52	41
	Female, *n* (%)	0	10 (19)	9 (22)
21	AIDS Patient Care and STDs	1	3	29
	Female, *n* (%)	0	1 (33)	10 (34)
22	Journal of Infectious Diseases	1	13	211
	Female, *n* (%)	1 (100)	5 (38)	56 (26)
23	International Journal of Infectious Diseases	1	3	26
	Female, *n* (%)	0	2 (66)	7 (27)
24	Current Opinion in Microbiology	2	0	40
	Female, *n* (%)	1 (50)	–	16 (40)
25	Reviews in Medical Virology	6	0	23
	Female, *n* (%)	0	–	6 (26)
26	Infectious Disease Modelling	2	5	30
	Female, *n* (%)	0	1 (20)	8 (26)
27	Travel Medicine and Infectious Disease	1	5	56
	Female, *n* (%)	0	1 (20)	20 (36)
28	Journal of the International AIDS Society	3	24	48
	Female, *n* (%)	2 (67)	18 (75)	26 (54)
29	Microbiology spectrum	1	332	9
	Female, *n* (%)	1 (100)	139 (42)	4 (44)
30	International Journal of Antimicrobial Agents	1	37	41
	Female, *n* (%)	0	8 (21)	8 (19)
31	Current HIV/AIDS Reports	1	17	20
	Female, *n* (%)	0	10 (58)	8 (40)
32	The Lancet Regional Health - Western Pacific	1	0	16
	Female, *n* (%)	1 (100)	–	10 (62)
33	Gut Microbes	1	28	49
	Female, *n* (%)	1 (100)	9 (32)	17 (34)
34	Journal of Virus Eradication	1	3	51
	Female, *n* (%)	1 (100)	3 (100)	19 (37)
35	Epidemics	3	1	36
	Female, *n* (%)	2 (67)	0	12 (33)
36	Infectious Diseases of Poverty	7	9	40
	Female, *n* (%)	1 (14)	2 (22)	9 (22)
37	Trends in Parasitology	1	0	0
	Female, *n* (%)	0	–	–
38	Journal of Microbiology, Immunology and Infection	5	71	0–
	Female, *n* (%)	0	16 (22)	
39	Biosafety and Health	4	10	64
	Female, *n* (%)	2 (50)	3 (30)	13 (20)
40	AIDS and Behavior	1	10	91
	Female, *n* (%)	0	6 (60)	41 (45)
41	Antimicrobial stewardship & healthcare epidemiology	2	5	23
	Female, *n* (%)	1 (50)	3 (60)	11 (47)
42	Infection control & hospital epidemiology	2	4	62
	Female, *n* (%)	0	3 (75)	25 (40)
43	American journal of infection control	1	9	43
	Female, *n* (%)	1 (100)	8 (88)	29 (67)
44	The journal of hospital infection	3	3	103
	Female, *n* (%)	0	1 (33)	30 (29)
	Total, *n*	84	839	1863
	Females, *n* (%)	22 (26)	305 (36)	585 (31)

* Ranking according to Scimago Journal & Country Rank. Numbers 41 to 44 are a convenience sample of hospital epidemiology journals.

The number of listed individuals per journal varied from 1 to 342. The categorization by decision level showed that 84 editorial members (3%) were at level I, 839 (30%) at level II, and 1863 (67%) at level III. Overall, 912 (33%) editors were assigned as females and 1,874 (67%) as males. The frequency distribution of the assigned gender by decision-making level shows that 22 (26%) females and 62 (74%) males were at level I, 305 (36%) females and 534 (64%) males at level II; and 585 (31%) females and 1,278 (69%) males at level III.

Several journals had some gender balance at all decision levels. For instance, in *Antimicrobial Stewardship & Healthcare Epidemiology* with 30 editors, females represented 50%, 60%, and 47% of the decision levels I to III, respectively; in *Journal of the International AIDS Society*, with 75 editorial members, females represented 67%, 75%, and 54% of decision levels I, II, and III, respectively. *Microbiology Spectrum* had 342 editors, and females composed 100%, 42%, and 44% of editorial members in decision levels I to III, respectively. *Current HIV/AIDS Reports* had 38 editors, and females composed 0, 58%, and 40% of editorial members in decision levels I to III, respectively. However, several others were unbalanced. For instance, in a journal (ranking 7, Table 1) with 15 members, no females occupied level I, 11% were at level II, and none were at level III. In another journal (ranking 12, Table 1), with 66 editors, females composed 0, 12%, and 26% at levels I to III, respectively. In the journal ranked 18 (Table 1), which had 105 editors, females composed 0 at decisions level I and II, and 16% of decision level III.

As expected, decision level I has few positions per journal, which prevents the further assessment of gender distribution. However, editors at decision level II were more numerous and still significantly influenced a journal. Table 1 shows that 29 journals had 4 or more editors at decision level II. Among these, 12 journals had less than 30% of females assigned to these positions, while 7 had 50% or more females.

We found 81 countries of affiliation for the 2,786 editors of the 44 journals. Table 2 displays the countries with 34 or more editors. The USA, United Kingdom, and China had editors in 41, 33, and 29 journals, respectively. Additionally, the share of editors from the USA, United Kingdom, and China represented 63% of the total in the assessed journals. One country (USA) had 1,276 editors, representing 46% of editors’ affiliations, although 21 countries had only 1 editor. Overall, 1 in every 4 editorial positions at the decision level I was occupied by a female, with the USA having the most females. Indeed, 64% of level I editors were from the USA. Once again, at decision level II, females affiliated with the USA accounted for more than half of these positions. We found no significant relationship between countries and the gender of the editors, at any decision level.

**Table 2.** Distribution of editorial members by gender in countries with 34 or more editors

		Level of decision					
Country^a^, number of editors	Journals involved	Level I		Level II		Level III	
		Women, *n* (%)	Men, *n* (%)	Women *n* (%)	Men, *n* (%)	Women, *n* (%)	Men, *n* (%)
Australia, *n* = 87	25	0	1 (100)	7 (29)	17 (71)	29 (47)	33 (53)
Canada, *n* = 96	26	0	1 (100)	13 (41)	19 (59)	23 (36.5)	40 (63.5)
China, *n* = 265	29	2 (17)	10 (83)	17 (19)	71 (81)	24 (14.5)	141 (85.5)
France, *n* = 72	21	0	0	13 (36)	23 (64)	5 (14)	31 (86)
Germany, *n* = 61	18	0	1 (100)	5 (38.5)	8 (61.5)	8 (17)	39 (83)
Italy, *n* = 47	21	0	0	9 (53)	8 (47)	10 (33)	20 (67)
Japan, *n* = 34	20	0	1 (100)	1 (33)	2 (67)	0	30 (100)
Netherlands, *n* = 40	19	0	3 (100)	5 (42)	7 (59)	8 (32)	17 (68)
South Africa, *n* = 40	15	0	0	4 (57)	3 (43)	10 (30)	23 (70)
Switzerland, *n* = 57	19	1 (33)	2 (67)	3 (21)	11 (79)	9 (22.5)	31 (77.5)
Taiwan, *n* = 83	9	0	5 (100)	19 (27)	52 (73)	1 (14)	6 (86)
UK, *n* = 216	33	3 (23)	10 (77)	14 (34)	27 (66)	60 (37)	102 (63)
USA, *n* = 1,276	41	14 (38)	23 (63)	154 (43)	202 (57)	320 (36)	563 (64)
Rest of the world, *n* = 412	36	2 (29)	5 (71)	41 (33)	84 (67)	78 (30)	202 (72)
Totals, *n* = 2786		22 (26)	62 (74)	305 (36)	534 (64)	585 (31)	1,278 (69)

a No significant associations between countries and the gender of the editors at any level of decision.

Table 3 displays the frequency distribution of male and female editorial members based on their decision-making levels, as well as the proportion of female and male first and last authors in the sample of 12 journals for articles published in 2019. Female authors comprised 50% of first authors and 36% of last authors. Our analysis shows weak but significant correlations between the editors’ gender and the gender of the first and last authors. The Pearson chi-square test for independence indicated that there was an association for some journals between the editor’s gender and decision level when the first author was female (χ2 = 82, df = 11, *P* < 0.001, Phi = 0.174).

**Table 3.** Gender distribution of the editorial members by decision level, first authors, and last authors of the original articles published in a sample of 12 journals

		Editors^b^			Authors Published Articles^b^				
		Level of decision, number editors, female (%)				First author, *n* (%)		Last author, *n* (%)	
Journal ranking	Journal name	I	II	III	Articles	Female	Male	Female	Male
3	The Lancet Infectious Diseases	1, 1 (100)	1, 0	25, 14 (56)	100	41 (41)	59 (59)	31 (31)	69 (69)
7	Journal of Infection	2, 0	9, 1 (11)	4, 0	98	44 (45)	54 (55)	32 (33)	66 (67)
8	Clinical Infectious Diseases	1, 0	23, 10 (43)	209, 52 (25)	572	265 (46)	307 (54)	193 (34)	379 (66)
12	Emerging Infectious Diseases	2, 0	25, 3 (12)	39, 10 (26)	302	150 (50)	152 (50)	93 (31)	209 (69)
16	Emerging Microbes and Infections	2, 0	6, 0	51, 10 (20)	147	51 (35)^a^	96 (65)^a^	40 (27)	107 (73)
20	Journal of Medical Virology	1, 0	52, 10 (19)	41, 9 (22)	331	145 (44)	186 (56)	103 (31)	228 (69)
22	Journal of Infectious Diseases	1, 1 (100)	13, 5 (38)	211, 56 (26)	474	248 (52)	226 (48)	155 (33)	319 (67)
28	Journal of the International AIDS Society	3, 2 (67)	24, 18 (75)	48, 26 (54)	157	99 (63)^a^	58 (37)^a^	88 (56)	69 (44)
29	Microbiology Spectrum	1, 1 (100)	332, 139 (42)	9, 4 (44)	111	47 (42)	64 (58)	40 (36)	71 (64)
31	Current HIV/AIDS Reports	1, 0	17, 10 (58)	20, 8 (40)	45	30 (67)^a^	15 (33)^a^	20 (44)	25 (56)
34	Journal of Virus Eradication	1, 1 (100)	3, 3 (100)	51, 19 (37)	28	16 (57)	12 (43)	8 (29)	20 (71)
40	AIDS and Behavior	1, 0	10, 6 (60)	91, 41 (45)	360	237 (66)^a^	123 (34)^a^	171 (47.5)	189 (52.5)
	Totals	17, 6 (35)	515, 205 (40)	798, 249 (31)	2725	1373 (50)	1352 (50)	974 (36)	1751 (64)

a Significant correlation between editors and first authors, when the first author was female (χ2 = 82, df = 11, *P* < 0.001, Phi = 0.174).

b Round numbers.

In the sample of 12 journals, first authors from 105 different countries published a total of 2,725 articles; 62% of the publications came from 5 countries, while the remaining 100 countries were grouped as “rest of the world” for comparison purposes (Table 4). The gender of the first author showed strong correlation with the country of affiliation (Cramér’s V 0.84, *P* = 0.002), whereas the gender of the last author and the country of affiliation had a weak correlation (Cramér’s V 0.08, *P* = 0.004). Our analysis did not show significant associations between authors’ gender and their chosen journals.

**Table 4.** Gender distribution of first and last authors by country in sample of 12 journals^a^

Country	Publications, *n* (%)	First author		Last author		Preferred journals, number of publications (%)
		Female, *n* (%)	Male, *n* (%)	Female, *n* (%)	Male, *n* (%)	
Australia	84 (3)	45 (54)	39 (46)	32 (38)	52 (62)	Clin Infect Dis, *n* = 16 (19) J Infect Dis, *n* = 16 (19) Emerg Infect Dis, *n* = 11 (13)
China	242 (9)	103 (43)	139 (57)	61 (25)	181 (75)	J Med Virol, *n* = 100 (41) Emerg Microbes Infect, *n* = 51 (21) J Infect Dis, *n* = 30 (12)
France	104 (4)	53 (51)	51 (49)	35 (34)	69 (66)	Clin Infect Dis, *n* = 22 (21) Emerg Infect Dis, *n* = 16 (15) J Infect Dis, *n* = 15 (14)
United Kingdom	193 (7)	88 (46)	105 (54)	72 (37)	121 (63)	Clin Infect Dis, *n* = 62 (32) J Infect Dis, *n* = 33 (17) Lancet Infect Dis, *n* = 22 (11)
United States of America	1,077 (39)	592 (55)	485 (45)	419 (39)	658 (61)	Clin Infect Dis, *n* = 274 (25) Aids Behav, *n* = 273 (25) J Infect Dis, *n* = 188 (17.5)
Rest of the world, *n* = 100 countries	1,025 (38)	492 (48)	533 (52)	355 (35)	670 (65)	J Infect Dis, *n* = 192 (19) Clin Infect Dis, *n* = 189 (18) J Med Virol, *n* =185 (18)
Totals, *n*=	2,725	1,373 (50)	1,352 (50)	974 (36)	1,751 (64)	

a Round numbers.

## Discussion

Diversity and equity are essential drivers for excellence and innovation, and these principles are equally vital for the advancement of science in all fields. However, it is evident that the editorial structure of ID and HE journals has not yet achieved diversity in terms of gender representation. Our study reveals that females are underrepresented at all decision levels within the editorial system. Only 26% serve in top leadership roles such as editors-in-chief or similar positions, 36% are in associate editor roles or similar positions, and 31% are part of the editorial boards. Strikingly, some journals exhibit a complete absence of females at the highest decision-making levels (I and II), and only a minority of females are present at level III.

This gender disparity aligns with trends observed in various medical specialties, as recently reported in leading medical journals,^9^ British Medical Journal Editorial Group journals,^19^ gastroenterology,^20^ medical education,^13^ healthcare simulation journals,^21^ ID,^10^ and rheumatology.^15^ For instance, Ayada et al. reported that only 22% of editors-in-chief in ID and microbiology journals were women, a figure similar to our findings, although the study utilized a different approach.^10^ Similarly, our results are in line with those reported for rheumatology, using the same methodology.^15^

These figures can be interpreted from different angles. One perspective would be that there might be intentional limitations on access to level I or level II editorial positions based on gender, akin to vertical segregation or the “glass ceiling” phenomenon. However, although our study was not designed to assess vertical segregation explicitly, the findings suggest that such a system does not exist within the editorship of ID and HE journals. This conclusion is supported by the fact that approximately half of the first authors of the reviewed articles were females, and the gender imbalance is not uniform across journals.

Another perspective would be that the ID and HE editors’ imbalance reflects what happens in other areas. This could be related to a self-perpetuating cycle, where practices and decisions remain unchanged for decades. The results of the exploratory variables studied here suggest country-related cultural, social, and organizational influences on editors’ gender composition, first and last authors’ gender, and the journal’s preference for where to publish. For instance, half of the 2,786 editors are affiliated with only 2 countries. On one side are the USA and the United Kingdom, where around 38% of the editors are females. On the other side are Japan and China, where 3% and 16% of editors at any decision level are females, a similar figure to what was found in rheumatology.^15^

Despite the dominance of two countries in publishing almost half of the articles we reviewed, the overall distribution of female and male authors is almost even for first authors, and 30% females and 70% men for last authors, consistent with other recent reports.^14,15,22^ Considering the growth of the ID workforce and the increasing number of older-than-55 ID specialists, it is plausible to consider that we are witnessing a generational transition. However, the cross-sectional nature of our study precludes making definitive conclusions in this regard, though external evidence supports this hypothesis.^13,14,23,24^

Our study identified a correlation between a higher proportion of female editors and increased publications with female first authors in some journals. Similar patterns were found in ID journals in 2018 and 2019^25^ and a microbiology journal in 2017,^26^ but this correlation was not observed in a rheumatology study employing a similar methodology in 2019.^15^ It is important to note that the magnitude of the correlation found in our study is relatively small, and the exploratory nature of our research precludes drawing definitive inferences.

While our study has several strengths, including the inclusion of a significant number of ID journals, the categorization of editors according to their level of influence, and the rigorous gender assignment methods, it also has several limitations. Primarily, the cross-sectional design restricts the study to only providing point prevalence and exploratory data, which may serve as a foundation for hypothesis generation and informing future data collection and research. Furthermore, our study focuses on reporting proportions of females and males but without establishing a specific threshold of a desirable balance. However, the clear gender imbalance we observed can serve as a basis for further examination of gender equality and equity^27^ within the editorial system of ID and hospital epidemiology journals. Additionally, our use of gender as a binary variable can be critiqued, as gender is a spectrum.^28^ However, we adopted this strategy due to data acquisition feasibility and its alignment with the approaches of other publications,^13,15^ as well as international institutions such as the World Bank^29^ and the United Nations Educational, Scientific, and Cultural Organization (UNESCO).^30^

In summary, our study shows that female editorial board members are underrepresented at all decision levels within the editorial system of ID and HE journals. Our findings suggest that the imbalance may be a result of long-standing practices and decisions perpetuating the underrepresentation of females. This study can serve as a baseline for monitoring the gender composition of editors and authors over time. It may also aid in reinforcing existing strategies implemented by IDSA^31^ and the Society for Healthcare Epidemiology of America (SHEA)^32^ or devising new approaches tailored to countries that significantly influence editors’ composition, with the goal of having a higher impact in a shorter timeframe. For instance, a training and development program, such as an IDSA-supported editorial fellowship for young ID physicians involving ID and HE journals, might be considered as a potential strategy. In the interim, fostering further discussion on representativeness, equality, and equity in ID and HE is essential and warranted.

## Data Availability

The data underlying this article will be shared on reasonable request to the corresponding author.
